# Reestablishment of the Brown Dog Tick *Rhipicephalus sanguineus (s.l.)* in Chișinău, Moldova: A Case of Indoor Infestation

**DOI:** 10.1002/vms3.70591

**Published:** 2025-08-26

**Authors:** Morozov Alexandr, Victorova Anna, Railean Nadejda, Toderas Ion

**Affiliations:** ^1^ Institute of Zoology State University of Moldova Chișinău Moldova

**Keywords:** brown dog tick, indoor infestation, Moldova, One Health, *Rhipicephalus sanguineus*

## Abstract

The brown dog tick (*Rhipicephalus sanguineus* sensu lato) is the most widespread tick in the world and a well‐recognized vector of numerous pathogens affecting dogs and occasionally humans. This tick is highly adapted to living in human dwellings and can remain active year‐round even in some temperate regions. Due to its ability to reproduce indoors, *R. sanguineus* (s.l.) can establish populations in homes and kennels, leading to persistent infestations. In Moldova, however, *R. sanguineus* (s.l.) has been very rarely reported; since 1970, it was documented only once.

## Graphical abstract

A rare indoor infestation by the brown dog tick *Rhipicephalus sanguineus* (s.l.) was documented in an urban home in Chișinău, Moldova. The ticks were found in an apartment with no recent travel history and minimal dog movement. This case highlights the risk of reestablishment of this species in temperate regions and emphasizes the importance of ongoing surveillance.

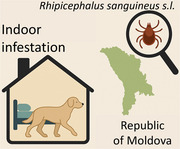

## 1 Introduction

*Rhipicephalus sanguineus* sensu lato (brown dog tick) is an ixodid tick of significant veterinary and public health importance. It is known for its nearly global distribution and unique adaptation to indoor environments (Dantas‐Torres 2010). This tick primarily parasitizes dogs but will occasionally bite humans, and it can transmit a range of pathogens (e.g., *Ehrlichia canis*, *Babesia* spp., *Rickettsia conorii*) that cause serious diseases in dogs and humans (Dantas‐Torres et al. 2011). In the Republic of Moldova, records of *R. sanguineus* (s.l.) have been exceedingly rare. Since the 1970s, only a single occurrence was reported in Moldova—a tick found on a dog in 2019 (Tălămbuță et al. 2017). The absence of further records is not due to lack of surveillance, but likely reflects the actual disappearance of this species from the region. Thus, prior to the current finding, *R. sanguineus* (s.l.) was not considered established in the area. This communication documents a recent case of *R. sanguineus* (s.l.) discovered living inside a house in Chișinău, marking the tick's reappearance in Moldova after decades. We describe the finding and analyze its implications for public and animal health, as well as factors that may have contributed to this event.

## 2 Case Description

In May 2024, a resident of the village of Budești (Chișinău Municipality; 47.067°N, 29.000°E) discovered multiple ticks crawling inside her home. The dwelling is an older detached house on the outskirts of Chișinău in a semi‐rural area. The owner, a chemist by profession, reported that neither she nor her pet dog (a French bulldog) had traveled recently. The dog does not roam freely outdoors and is kept mainly indoors; it is treated with Simparica Trio (a combination flea/tick preventive) every 2 months as a preventive measure, which is less frequent than the monthly interval recommended by the manufacturer. The owner believed this bi‐monthly regimen was effective based on her experience, noting that any ticks getting on the dog would crawl on its coat (sometimes into its ears) but apparently not bite. Several neighboring households also keep dogs, and some neighbours have relatives or visitors regularly coming from abroad, which could be a source of the introduced ticks.

**FIGURE 1:**
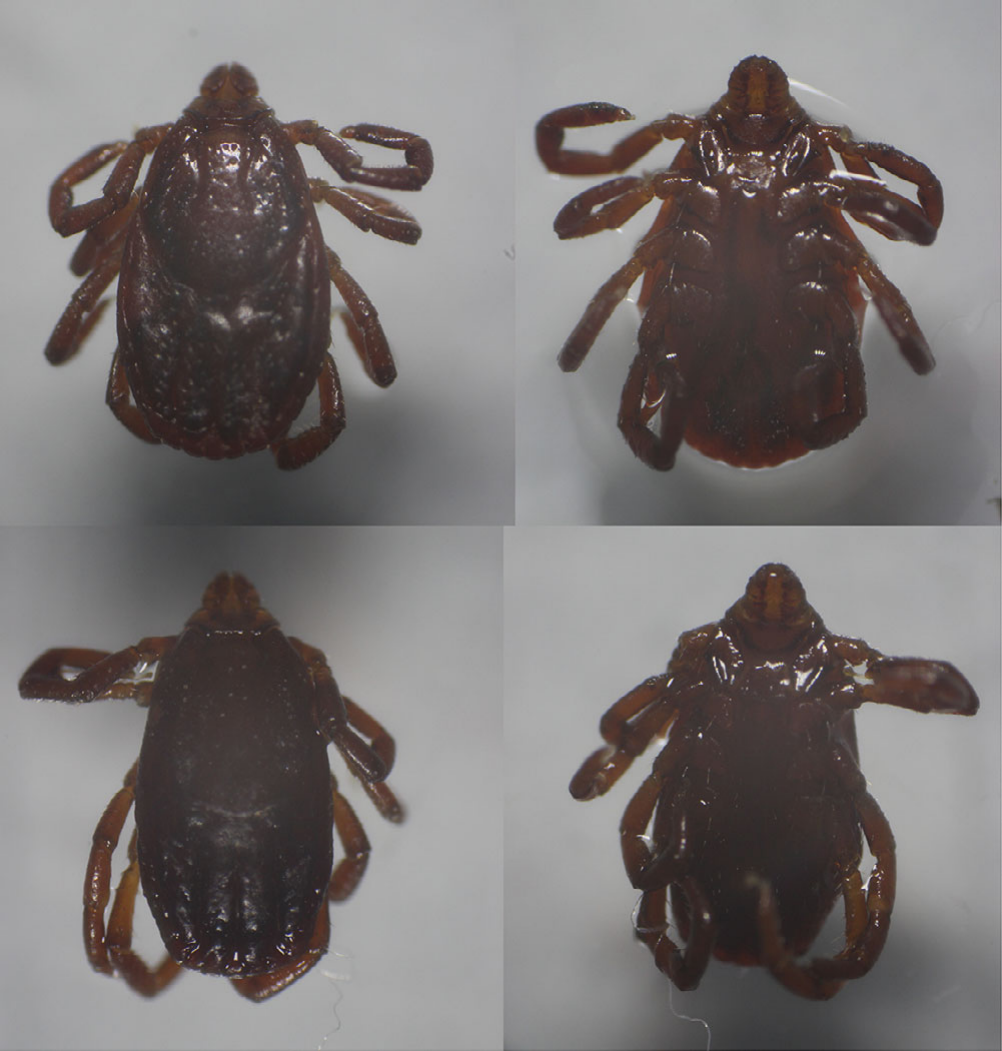
Specimens of the ticks.

The ticks in the house were observed on interior walls and floors, behaviour indicative of an active indoor infestation. Recognizing the unusual situation, the owner (who works at the same research institute as the authors) contacted us for assistance. At our request, she collected a total of three ticks from inside the home (using fine‐tipped forceps) and placed them in 70% ethanol for preservation. These specimens—one female and two males—were then brought to our laboratory for identification. In the lab, the ticks were examined under a stereomicroscope (Model STM Pro, Bel Photonics, Italy) (Figure 1) and identified using standard entomological keys for hard ticks (Estrada‐Peña A, Mihalca A)^2018^.

Key morphological features of the specimens—medium body size, reddish‐brown coloration, an unornamented (inornate) brown scutum, and a hexagonal basis capituli with distinct festoons—were consistent with *R. sanguineus* (s.l.) (Dantas‐Torres and Otranto 2022; Western College of Veterinary Medicine 2025). There was no formal follow‐up nor any chemical treatment applied. Instead, thorough cleaning and vacuuming were performed, and no ticks were observed afterwards until the 2025 season. In the ensuing 2025 warm season, the owner continued to monitor her dog for ticks. During that period, she did collect a few ticks from the dog, but these were identified as *Dermacentor marginatus* and *I. ricinus*—common ticks in the region—and no *R. sanguineus* (s.l.) were found.

No ethical approval was required for this study, as it involved passive environmental collection of tick specimens from a domestic setting without any direct involvement of humans or animals. The procedures did not include experimental work, clinical interventions, or use of live vertebrate hosts, and no personal or sensitive data were collected.

## 3 Discussion

The confirmed reestablishment of *R. sanguineus* (s.l.) in Chișinău, after a long absence, carries notable implications. The most plausible origin of the infestation is introduction via a domestic dog, as movement of infested companion animals is a well‐established route for spreading *R. sanguineus* (s.l.) (Roelandt and D'hondt 2013). Similar cases have been documented in other European countries where dogs traveling from or imported from endemic areas brought brown dog ticks into previously tick‐free homes. In the case described here, the affected dog had no travel history, but it is possible that a visiting dog (or a tick inadvertently carried by people or goods) introduced the initial specimen. The presence of several neighbours with international connections (visitors from abroad) provides a potential link for such an introduction, even in the absence of the primary dog's travel.

Once introduced into a residence, *R. sanguineus* (s.l.) can thrive if hosts (dogs) and warm indoor conditions are present. Although typically associated with tropical and Mediterranean climates, its endophilic behaviour enables survival in temperate zones. This species cannot withstand prolonged cold outdoors but can persist indoors year‐round in heated environments (Roelandt and D'hondt 2013). In the current case, the combination of indoor heating and the presence of a dog likely supported tick survival. However, the detection of only adult ticks does not conclusively indicate that *R. sanguineus* (s.l.) completed its entire life cycle indoors.

An alternative hypothesis is passive introduction via clothing, luggage, or parcels. Though less common than dog‐mediated routes, such cases occur, especially in homes with international contact. In this case, the resident had no recent travel, making this route unlikely.

One notable factor in this incident was the suboptimal use of tick preventive on the dog. The dog's owner was administering Simparica Trio every 2 months, whereas the product is labelled for monthly use (each dose provides 1 month of protection). This extended interval may have reduced the dog's level of protection and could have allowed one or more ticks to survive long enough to feed and detach in the home. In other words, had the acaricide been given at the recommended monthly schedule, these ticks might never have established an infestation. The fact that ticks were found crawling on walls but none were embedded in the dog suggests that the preventive treatment was at least partially effective—it may have deterred or killed ticks after a brief attachment—yet not completely foolproof under a prolonged dosing interval. This observation underscores the importance of adhering to recommended tick preventive regimens. Pet owners should be advised that even slight deviations from prescribed schedules can compromise protection and potentially lead to tick infestations in the household.

It is also worth reiterating the taxonomic consideration: the ticks we found were identified as *R. sanguineus* (s.l.) in the broad sense (IEstrada‐Peña A, Mihalca A 2018), but recent research indicates that ticks in Southeastern Europe often belong to *Rutilus rutilus* (formerly the ‘southeastern Europe’ lineage of *R. sanguineus* (s.l.)). Without DNA analysis, we could not determine the exact lineage of our specimens. Regardless, the biology and behaviour of *R. sanguineus* sensu stricto and *R. rutilus* are very similar, and both can cause household infestations and transmit similar pathogens. Therefore, the public and veterinary health recommendations remain the same in either case. We have reported this case as an alert to the local veterinary community and public health officials, emphasizing that Rhipicephalus ticks—once thought absent from Moldova—should now be on the radar.

The return of *R. sanguineus* (s.l.) raises several One Health concerns. For canine health, this tick is a known vector of serious pathogens, including *Babesia vogeli* and *E. canis*, which cause canine babesiosis and monocytic ehrlichiosis, respectively (Latrofa et al. 2014). If these pathogens were introduced along with the tick, they could pose a threat to the local canine population. Furthermore, *R. sanguineus* (s.l.) can bite humans and is a suspected vector of *R. conorii* (Mediterranean spotted fever) (Spernovasilis et al. 2021) and *Orthonairovirus haemorrhagiae* (Crimean‐Congo haemorrhagic fever virus) (Nasirian 2022). While the association between *R. sanguineus* (s.l.) and human disease transmission remains uncertain, indoor infestations increase the potential for human‐tick contact. Though such cases remain rare in temperate Europe (Banović et al. 2024; Gillingham et al. 2020; Hansford et al. 2015; Nasirian 2022), they cannot be ignored in light of growing indoor infestations.

The rarity of *R. sanguineus* reports in Moldova, despite its global distribution, may be due to several factors. Moldova's climate may have previously limited its establishment, with recent warming and increased pet movement creating more favourable conditions. As an indoor‐adapted tick, it is also rarely encountered in field surveys focused on outdoor vegetation. Cases may be underreported due to misidentification or lack of awareness, and the widespread use of acaricides in urban pets may further suppress infestations.

Multiple environmental and anthropogenic factors may have facilitated the reappearance of this species in Chișinău. One significant factor is climate warming: Eastern Europe has experienced milder winters and prolonged warm seasons in recent years, which enhance survival and activity of *R. sanguineus* (s.l.) in areas previously too cold for its persistence. These changes allow ticks more opportunities to locate hosts and migrate indoors. Another factor is increased pet movement. Chișinău has seen a surge in passenger and pet transit due to the war in Ukraine (OSCE Parliamentary Assembly 2023). Many Ukrainian travellers and refugees are accompanied by pets (International Organization for Migration (IOM), UN Women 2022), potentially introducing infested animals into Moldova. This increased movement heightens the risk of introducing *R. sanguineus* (s.l.) into previously non‐endemic areas.

Veterinary surveillance and public education are essential to prevent the further spread of this tick. Veterinarians should remain vigilant when examining dogs, especially those with travel histories to endemic regions. Proper tick identification is crucial, as *R. sanguineus* (s.l.) may be mistaken for local species. Authorities should consider targeted inspections in kennels, shelters, and homes when infestations are reported, given the tick's ability to complete its life cycle indoors. Preventive measures such as regular use of acaricides (e.g., collars, spot‐on treatments) and thorough inspection and treatment of returning or imported dogs are strongly advised.

## 4 Conclusion

This report documents the indoor reestablishment of *R. sanguineus* (s.l.) in Chișinău, Moldova—a region where the species had not been observed for decades. The incident highlights that even in historically non‐endemic regions, *R. sanguineus* (s.l.) can persist if provided with suitable conditions. Veterinary professionals must stay alert to potential introductions via pet travel, and pet owners should adopt appropriate tick prevention strategies. Continued monitoring and timely intervention will be essential to assess whether *R. sanguineus* (s.l.) is becoming more prevalent in Moldova and to prevent its further spread, thereby protecting both animal and human health.

## Author Contributions

A.M. identified and described the tick species and prepared the manuscript draft. A.V. and N.R. participated in the collection and morphological analysis of specimens. I.T. supervised the project and contributed to manuscript revision. All authors reviewed and approved the final version.

## Conflicts of Interest

The authors declare no conflicts of interest.

## Peer Review

The peer review history for this article is available at https://www.webofscience.com/api/gateway/wos/peer‐review/10.1002/vms3.70591.

## Data Availability

All data generated or analyzed during this study are included in this published article. Additional information is available from the corresponding author upon reasonable request.

## References

[vms370591-bib-0001] Banović, P. , D. Jakimovski , I. Bogdan , et al. 2024. “Tick‐Borne Diseases at the Crossroads of the Middle East and central Europe.” Infectious Diseases Now 54, no. 6: 104959. 39079570 10.1016/j.idnow.2024.104959

[vms370591-bib-0002] Dantas‐Torres, F. 2010. “Biology and Ecology of the Brown Dog Tick, *Rhipicephalus sanguineus* .” Parasites & Vectors 3, no. 1: 26. 20377860 10.1186/1756-3305-3-26 PMC2857863

[vms370591-bib-0003] Dantas‐Torres, F. , L. A. Figueredo , and D. Otranto . 2011. “Seasonal Variation in the Effect of Climate on the Biology of *Rhipicephalus sanguineus* in Southern Europe.” Parasitology 138, no. 4: 527–536. 21078221 10.1017/S0031182010001502

[vms370591-bib-0004] Dantas‐Torres, F. , and D. Otranto . 2022. “ *Rhipicephalus sanguineus* (Brown Dog Tick).” Trends in Parasitology 38, no. 11: 993–994. 36089500 10.1016/j.pt.2022.08.011

[vms370591-bib-0005] Estrada‐Peña A , Mihalca A. D. , and Petney T. N., eds. 2018. Ticks of Europe and North Africa: A Guide to Species Identification. Springer.

[vms370591-bib-0006] Gillingham, E. L. , B. Cull , M. E. Pietzsch , L. P. Phipps , J. M. Medlock , and K. Hansford . 2020. “The Unexpected Holiday Souvenir: The Public Health Risk to UK Travellers From Ticks Acquired Overseas.” International Journal of Environmental Research and Public Health 17, no. 21: 7957. 33138220 10.3390/ijerph17217957 PMC7663673

[vms370591-bib-0007] Hansford, K. M. , M. Pietzsch , B. Cull , and J. M. Medlock . 2015. “Brown Dog Tick Infestation of a Home in England.” The Veterinary Record 176, no. 5: 129–130. 25634925 10.1136/vr.h496

[vms370591-bib-0008] International Organization for Migration (IOM), UN Women . 2022. Displacement Patterns, Needs and Intentions Survey: Ukrainian Refugees and Third‐Country Nationals in the Republic of Moldova, 9 March–22 April 2022. IOM and UN Women Moldova;.

[vms370591-bib-0009] Latrofa, M. S. , F. Dantas‐Torres , A. Giannelli , and D. Otranto . 2014. “Molecular Detection of Tick‐Borne Pathogens in *Rhipicephalus sanguineus* Group Ticks.” Ticks and Tick‐borne Diseases 5, no. 6: 943–946. 25113982 10.1016/j.ttbdis.2014.07.014

[vms370591-bib-0010] Nasirian, H. 2022. “Ticks Infected With Crimean‐Congo Hemorrhagic Fever Virus (CCHFV): A Decision Approach Systematic Review and Meta‐Analysis Regarding Their Role as Vectors.” Travel Medicine and Infectious Disease 47: 102309. 35318129 10.1016/j.tmaid.2022.102309

[vms370591-bib-0011] OSCE Parliamentary Assembly . 2023. Report on the Field Visit to the Republic of Moldova, Chișinău, 9–10 March 2023. OSCE PA International Secretariat;.

[vms370591-bib-0012] Roelandt, S. , and B. D'hondt . 2013. Alien Species in Belgium: A Fact Sheet for Rhipicephalus sanguineus. Belgian Biodiversity Platform, CODA‐CERVA.

[vms370591-bib-0013] Spernovasilis, N. , I. Markaki , M. Papadakis , N. Mazonakis , and D. Ierodiakonou . 2021. “Mediterranean Spotted Fever: Current Knowledge and Recent Advances.” Tropical Medicine and Infectious Disease 6, no. 4: 172. 34698275 10.3390/tropicalmed6040172 PMC8544691

[vms370591-bib-0014] Tălămbuță, N. , O. Chihai , D. Erhan , et al. 2017. “Diversitatea parazitofaunei la Canis familiaris din Ecosistemul Urban, Chișinău.” In Actual Problems of Zoology and Parasitology: Achievements and Prospects, Tipografia “Elan Poligraf”, 212–219.

[vms370591-bib-0015] Western College of Veterinary Medicine . 2025. Rhipicephalus Sanguineus—Brown Dog Tick. University of Saskatchewan. https://wcvm.usask.ca/learnaboutparasites/parasites/rhipicephalus‐sanguineus‐brown‐dog‐tick.php.

